# Charged giant unilamellar vesicles prepared by electroformation exhibit nanotubes and transbilayer lipid asymmetry

**DOI:** 10.1038/s41598-018-30286-z

**Published:** 2018-08-07

**Authors:** Jan Steinkühler, Philippe De Tillieux, Roland L. Knorr, Reinhard Lipowsky, Rumiana Dimova

**Affiliations:** 1 Department of Theory and Bio-Systems, Max Planck Institute of Colloids and Interfaces, Science Park Golm, 14424 Potsdam, Germany; 2 Present Address: Department of Electrical Engineering, Polytechnique Montreal, Montreal, Quebec, H3T 1J4 Canada

## Abstract

Giant unilamellar vesicles (GUVs) are increasingly used as a versatile research tool to investigate membrane structure, morphology and phase state. In these studies, GUV preparation is typically enhanced by an externally applied electric field, a process called electroformation. We find that upon osmotic deflation, GUVs electroformed from charged and neutral lipids exhibit inward pointing lipid nanotubes, suggesting negative spontaneous curvature of the membrane. By quenching a fluorescent analog of the charged lipid, zeta potential measurements and experiments with the lipid marker annexin A5, we show that electroformed GUVs exhibit an asymmetric lipid distribution across the bilayer leaflets. The asymmetry is lost either after storing electroformed GUVs at room temperature for one day or by applying higher voltages and temperatures during electroformation. GUVs having the same lipid composition but grown via gel-assisted swelling do not show asymmetric lipid distribution. We discuss possible mechanisms for the generation and relaxation of lipid asymmetry, as well as implications for studies using electroformed vesicles. The observed effects allow to control the molecular assembly of lipid bilayer leaflets. Vesicle tubulation as reported here is an example of protein-free reshaping of membranes and is caused by compositional lipid asymmetry between leaflets.

## Introduction

Lipid bilayer sheets in aqueous environments can deform and close into vesicles to shield free edges from water. In general, vesicle formation can proceed spontaneously, i.e. by osmotic swelling. Under the right conditions, some vesicles can be *giant*, ranging in size from a couple of micrometers up to over several dozens of micrometers with one single closed lipid bilayer sheet. Giant unilamellar vesicles (GUVs) are used for a multitude of applications, from basic membrane research to synthetic biology^1–4^. To increase the yield, to speed up the process, and to reduce defects of such GUVs, several protocols have been proposed, see e.g. ref.^2^. Probably the most popular is the method of electroformation, in which GUV growth is accelerated by an external electrical field. Originally developed for one-component lipid bilayers in sugar solutions^5^, the protocol was quickly adapted for various complex lipid mixtures, buffers and even for protein reconstitution, see e.g.^2,6–8^. Often, it is implicitly assumed that as long as GUVs grow, they will represent a well equilibrated, defect-free system of the desired membrane composition. However, careful investigation of the electroformation method has revealed possible artefacts. For example, lipid oxidation can result from charge transfer from the electrode^9–11^. Also, aging of the commonly used indium tin oxide (ITO) coated glass electrodes seems to degrade the quality and size of GUVs^12^. It is also known that lipid compositions for vesicles in the same batch can vary, especially for multicomponent vesicles close to lipid demixing^13–15^.

In this work we focus on the difference in lipid composition between the two membrane leaflets. Considering the involvement of electrostatic interactions in the electroformation protocol, we questioned the effect it might have on the distribution of charged lipids across the membrane of electroformed GUVs. Asymmetric distribution of the lipid species in the two leaflets can have two major consequences. First, the composition and charge of the two leaflets will differ from the one for an (assumed) symmetric distribution. This asymmetry will affect studies addressing the binding of species such as proteins, peptides, and (nano) particles, and will obscure the comparison with results obtained on symmetric systems. Second, the asymmetry will lead to nonzero spontaneous curvature of the membrane. Thus, GUV-based assays assessing curvature sensing and generation will also be affected^16–19^.

Apart from advancing the understanding of GUV electroformation, membrane asymmetry (even though mostly undesired or unanticipated in model membrane studies) can be turned into an advantage for guiding and controlling the supramolecular (self-) assembly and organization of charged lipids at the nanometer scale of lipid membrane leaflets. Our study also demonstrates membrane reshaping by compositional bilayer asymmetry, a feature ubiquitous in biological membranes.

## Results and Discussion

### Morphologies of asymmetric DOPC/DOPG vesicles

Giant vesicles made of DOPC/DOPG (8/2 molar ratio) and pure DOPC were prepared via the standard electroformation protocol in HEPES buffer, see Material and Methods. Electroformation of GUVs proceeds by first depositing a thin lipid film on a conductive electrode, followed by evaporation of the organic solvent (usually chloroform or other non-polar solvents). Here we used ITO electrodes; similar results were obtained for GUVs grown on platinum wires, see below. The lipids on the electrodes were hydrated and a weak (<1 kV/m) AC-electric field was applied. We harvested the GUVs and inspected them directly under a confocal microscope and in symmetric HEPES buffer conditions across the membrane. The observed vesicle morphologies differ significantly between pure DOPC and DOPC/DOPG GUVs. For the majority of DOPC/DOPG vesicles, we found inward-pointing protrusions, such as buds and tubes, and the vesicles appeared mostly spherical without any optically resolvable membrane fluctuations, indicating high membrane tension. After 26 hours of storage, the majority of protrusions disappeared and GUVs often exhibited optically resolvable membrane fluctuations, indicating nearly vanishing membrane tension. In the case of pure DOPC lipids, most GUVs were free of any protrusions already after preparation while the remaining GUVs showed no preference for either inward or outward structures, see Table S1 in the Supporting Information (SI). Because, in symmetric buffer conditions, GUVs are floating freely in the observation chamber, quantitative characterization of the vesicle fraction with inward pointing structures is difficult.

**Figure 1:**
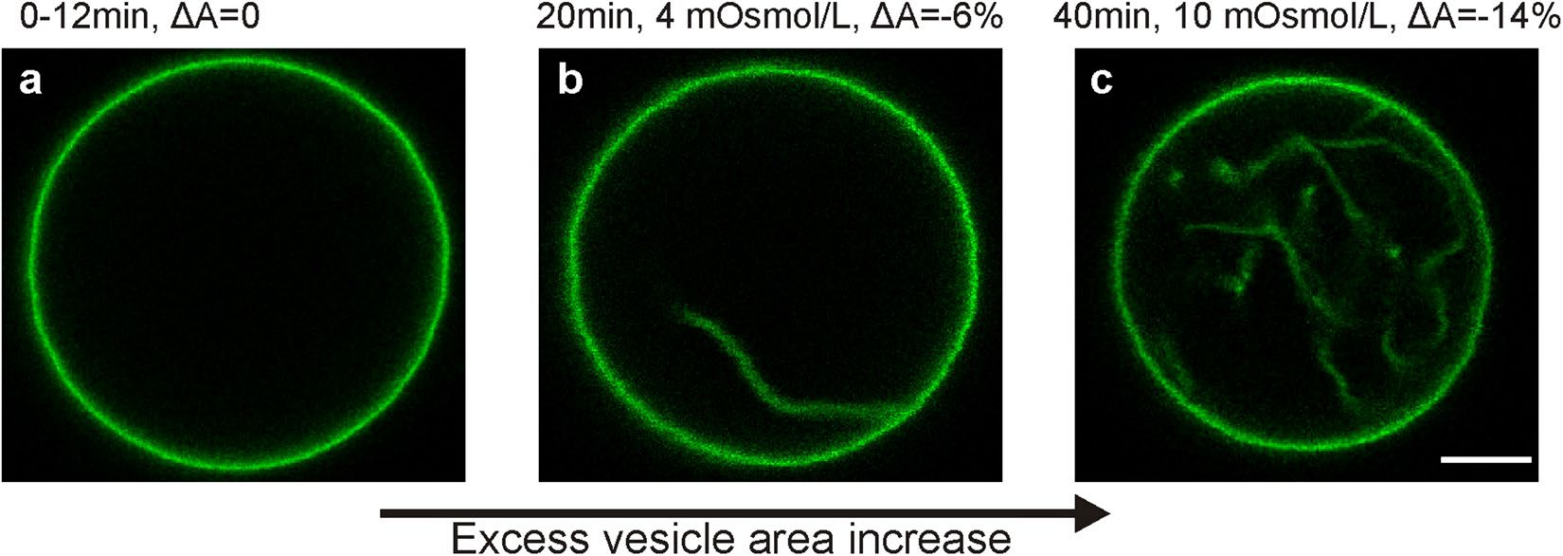
Deflation trajectory of a single DOPC/DOPG GUV with composition 8/2 (equatorial confocal cross sections) from (**a**) slightly hypotonic to (**b**,**c**) strongly hypertonic conditions achieved by evaporation. The text above the images indicates the time evolution since the beginning of the experiment, the osmolarity increase compared to the initial osmolarity of about 57 mOsmol/l, and the associated area change, ΔA = (A_app_ − A_0_)/A_0_, where A_app_ is the apparent area of the mother vesicle and A_0_ the initial area of the spherical GUV at the beginning of the deflation process. This excess area is stored in membrane tubes as seen in (**b**,**c**). Scale bar 5 μm.

To study the morphology of the inward protrusions, we controlled the osmotic conditions and electroformed DOPG/DOPC vesicles in the presence of sugars. The encapsulation of sucrose inside the vesicle and the presence of a sucrose/glucose asymmetry inside/outside has the benefit of settling the vesicles to the bottom of the observation chamber. Initially, the outside solution was made slightly hypotonic resulting in spherical vesicles without any visible membrane fluctuations (Fig. 1a). The purpose of this initial inflation step was to pull out membrane folds, similarly to the approach in micropipette aspiration experiments where vesicles are pre-stressed before measurements^20^. When the osmolarity of the outside solution was then slowly increased by evaporation, tubes appeared directed towards the vesicle interior in more than 90% of the GUVs (Fig. 1b,c). By following individual vesicles along their deflation trajectory, we found that the diameter of the tubes is always below the resolution of confocal imaging (<200 nm). We also observed that tubes grow in length, consistent with predicted trajectories for slow vesicle deflation^21^. Furthermore, molecules dissolved in the surrounding aqueous solution could freely access the interior of the tubes, which confirms the morphology of nanotubes filled with external solution and directed to the interior of the GUV, see Figure S1 in the SI. When vesicles were deflated in the same fashion after 26 hours of storage, we observed either defect-free floppy vesicles or vesicles with much thicker (optically resolvable) tube diameters, see Figure S2 in the SI.

The appearance of nanotubes in DOPC/DOPG GUVs depends on the specific preparation method. When gel-assisted swelling on polyvinyl alcohol (PVA) films^22^ was used to grow DOPC/DOPG GUVs in the absence of an electric field (see Material and Methods), we observed no preference for inward-pointing protrusions, see Figure S3 in the SI. This is similar to the behavior of pure, uncharged, DOPC GUVs prepared by electroformation, see Table S1 in the SI. We conclude that the observed tubulation does not represent a universal behavior of the DOPC/DOPG system, but is specific to electroformed DOPC/DOPG GUVs.

### DOPC/DOPG GUVs exhibit asymmetric lipid distribution when prepared by electroformation

The appearance of nanotubes in GUVs is a consequence of membrane asymmetry, which induces preferred (or spontaneous) curvature in bilayers^23^. The nature of the asymmetry can originate either from the compositional difference of the solutions on both sides of the membrane, or from the differences between the bilayer leaflets which may differ in their composition and/or their molecular densities leading to different molecular areas (bilayer asymmetry arising from different lipid densities or molecular areas has been studied by molecular simulations in ref.^24^). This in turn can itself be influenced e.g. by binding or adsorption of other molecules. Spontaneous tubulation in GUVs is a signature of nonzero spontaneous curvature. The sign convention assigns negative or positive values to protrusions (tubes, buds) pointing inwards or outwards, respectively, e.g., inward nanotubes imply negative spontaneous curvature^23^. Tubulation in GUVs has been previously reported as a result of asymmetric binding of proteins^16,25^, adsorption of polymers or pH-induced asymmetry in the headgroup area^18,21,26,27^. In the system studied here, neither adsorption/binding to the membrane nor changes in headgroup hydration are expected. We thus speculated that the observed morphologies result from lipid asymmetry between leaflets and investigated the distribution of DOPC and DOPG between the leaflets by fluorescent quenching experiments, see Material and Methods.

**Figure 2:**
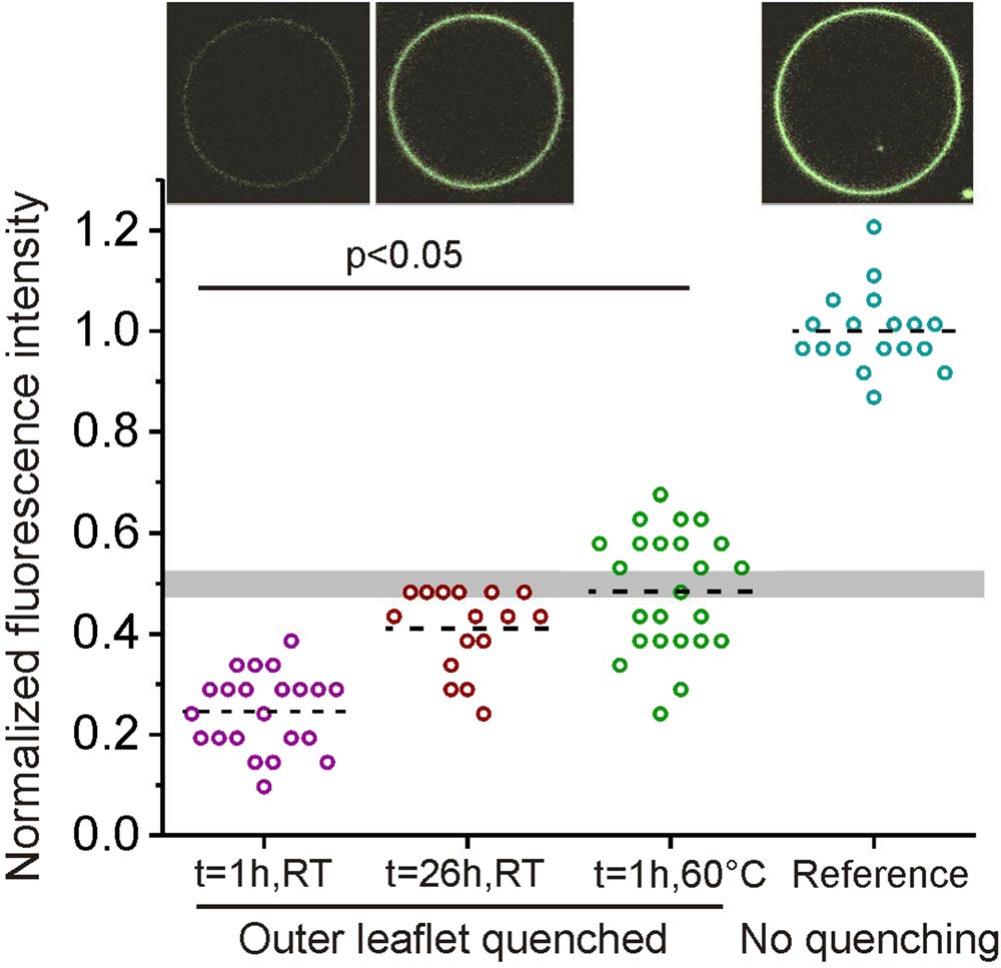
Fluorescent intensities before and after irreversible quenching of NBD-PG in the outer bilayer leaflet for different populations of 8/2 DOPC/DOPG vesicles. From left to right: Quenching 1 h after electroformation at room temperature (RT), quenching after 26-hour storage at RT, 1 h after electroformation at elevated temperature and voltage (see text for details) and reference value of unquenched vesicles at RT. The dashed lines indicate mean values. The fluorescence intensity was normalized by the mean value of the reference measurement. The gray bar is a guide to the eye and indicates half of the mean value (and the respective standard deviation) of the reference sample. Each data point represents one vesicle. All populations are significantly different from each other (Student t-test). Inserts show unedited snapshots of GUVs (green indicates NBD fluorescence) from the populations at t = 1 h and 26 h at RT as well as for the reference measurement corresponding to the data below each of the images. All GUVs are between 20 and 25 μm in diameter.

If (labeled) molecules in a membrane are symmetrically distributed between the two constituting bilayer leaflets, their concentration in each leaflet should be half of the total concentration, and quenching of a probe in the outer leaflet should decrease the fluorescence signal by half. When NBD-PG fluorescence of the outer membrane leaflet of electroformed DOPC/DOPG GUVs was quenched irreversibly by dithionite (to which the membrane is impermeable), the vesicles appeared almost dark having fluorescence intensity far below 50% of the original value, see Fig. 2. For each condition, the scatter in the data presumably results from the variation of the lipid compositions between vesicles of the same batch^13–15^). Since NBD-PG and DOPG have identical head groups (see Figure S4 in the SI), the data indicates preferential DOPG partitioning to the outer leaflet. When the same experiment was conducted on DOPC/DOPG vesicles stored for 26 hours at room temperature (RT) after electroformation, the average intensity is closer to 50% of the original fluorescence signal (Fig. 2). This observation suggests that the initially asymmetric NBD-PG distribution equilibrates over the time course of 26 hours, presumably caused by lipid trans-bilayer reshuffling via lipid flip-flops between the two bilayer leaflets. Indeed, the typical flip-flop time scales for PC lipids in closed vesicles are t_1/2_ ≈ 7 h^28^; we are not aware of studies for PG flip-flop half times in a comparable system.

To corroborate our observations and exclude possible artefacts resulting from the additional component introduced in the membrane, namely, the fluorescent lipid, we conducted zeta potential measurements on NBD-PG-free GUVs. In these experiments, we intended to use the surface charge as a probe of membrane asymmetry, see Material and Methods and Carvalho *et al*.^29^ for details and caveats of zeta potential measurements on GUVs. We found that the apparent zeta potential of GUVs decreases from −83 ± 10 mV to −61 ± 6 mV after 26 hour storage at RT. This result confirms our conclusion of an enrichment of DOPG in the outer leaflet of freshly electroformed vesicles, as found in quenching experiments.

Assuming an equal number of lipids in the two leaflets, the *inward* tubulation observed upon deflation of fresh electroformed GUVs is also consistent with the smaller area per lipid of DOPG molecules compared to that of DOPC^30,31^. The area mismatch between the leaflets can be relaxed when the membrane bulges towards the vesicle interior, thereby forming membrane segments with negative mean curvature such as inward pointing nanotubes^23^. For a more detailed molecular understanding of how negative spontaneous curvature is generated in asymmetric DOPG/DOPC bilayers, the lateral bilayer stress profile could be obtained, e.g. by computer simulations^24^.

We probed whether the AC-field electroformation conditions have an effect on the bilayer asymmetry. Indeed, an increase in electroformation voltage (from 0.6 V_rms_ to 2.2 V_rms_) and temperature (from RT to 60 °C) during the electroformation led to an essentially equal DOPG/DOPC composition in both leaflets, see Fig. 2, green data points. Interestingly, one has to increase both the voltage and the temperature during electroformation in order to suppress the asymmetric distribution of DOPG (data not shown). By which mechanism the increase in temperature and voltage lead to more symmetric distribution of the charged lipids is studied further below. Generally, one should be aware that electroformation conditions at very high voltages may lead to lipid oxidation^32^.

### Asymmetric membranes produced by alternative protocols

To extend our studies and to corroborate the general application of our findings to other systems and preparation protocols, we conducted experiments on membranes with another charged lipid, DOPS, of varying content (5, 10 and 20 mol%) and also performed electroformation on platinum electrodes instead of ITO glasses. Both DOPC/DOPS GUVs grown on ITO plates and DOPC/DOPG GUVs grown on platinum wires (see Material and Method and Figure S7 in the SI) consistently showed inward protruding nanotubes upon osmotic deflation in glucose solution of freshly prepared vesicles.

In addition, we tested the membrane asymmetry with a lipid specific probe. Being water soluble, but membrane impermeable, the protein annexin A5 binds PS lipids with high affinity and this binding is proportional to PS content up to about 20% PS^33,34^. DOPC GUVs doped with 5% DOPS were grown via electroformation and incubated with fluorescently labeled annexin A5 right after preparation (t = 1 h) or after 25 h of storage at room temperature. Incubation was performed in conditions mimicking physiological salt solutions with the addition of 2 mM CaCl_2_ (see Material and Methods). Annexin A5 binding was significantly reduced over time (by about 24%, see Figure S8). This observation is consistent with an initially asymmetric DOPS distribution that is equilibrated via flip-flops, leading to a reduction of DOPS at the outer vesicle leaflet available for binding annexin A5. Interestingly, the tubes formed in DOPS-doped membranes persisted for longer times compared to DOPG-doped vesicles, which could be a result of the slower flip-flop and/or the smaller lipid area of DOPS compared to that of DOPG^30,31^. Note that vesicles with a lower fraction of DOPS showed a reduced tendency to exhibit tubes with time: the majority of vesicles with 5 mol% DOPS did not form tubes after 25 h, while the majority of vesicles with 20 mol% DOPS still exhibited tubes after this time period.

Note that the above set of experiments with annexin A5 was performed with salt and sugar asymmetry across the membrane. The tube formation and the decrease in annexin A5 binding suggests that the compositional membrane asymmetry in fresh vesicles persists independently of the exact solution conditions. However, nanotube generation depends on the overall asymmetry across the membrane, i.e. both on the leaflet compositional asymmetry and on the asymmetry of the solutions on both sides of the membrane^21,35–37^, which can even influence the phase state of the membrane^38,39^. Sodium and sugar asymmetry across a membrane can lead to the generation of nonzero spontaneous curvature demonstrated in^40^ for sugar asymmetry. Additionally, asymmetric CaCl_2_ distributions also lead to the generation of membranes tubules^41^. Decoupling of the different contributions remains a challenge.

### Mechanisms for generation of charged lipid asymmetry

The generation of bilayer asymmetry requires different forces acting on the two leaflets on the membrane. In the following, we discuss conditions and forces that might induce or oppose asymmetric distribution of the lipids during electroformation, explored by further experiments.

#### Bilayer defects

**Figure 3:**
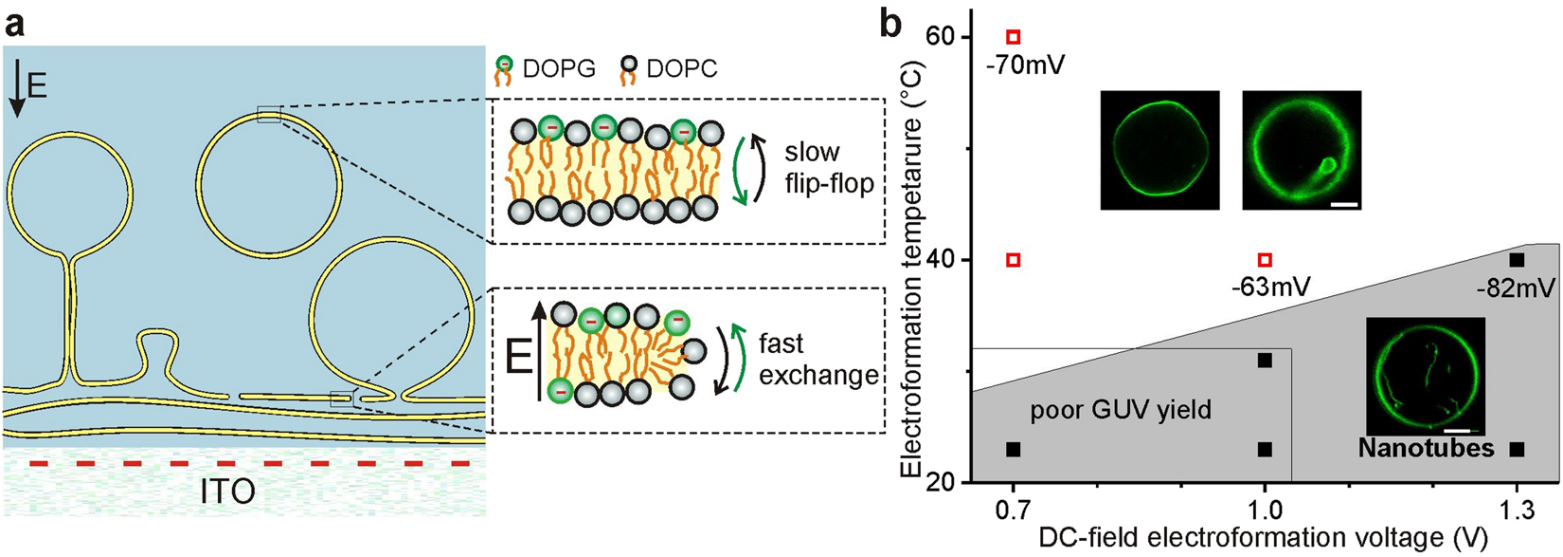
(**a**) Proposed mechanisms for the generation of bilayer asymmetry during vesicle electroformation. Surface affinity and electric field E act to sort negatively charged lipids (shown with green head groups) via transport through bilayer defects (edges or pores). Due to the absence of stable defects in closed GUVs, lipid redistribution after electroformation is slow and occurs only via flip-flop. The red dashed line indicates negative electrostatic charges on the bilayer and ITO surface. (**b**) DC-field electroformation conditions and morphology of deflated DOPC/DOPG GUVs. Solid black squares (data) and gray area (guide to the eye) indicate conditions at which more than 90% of the GUVs were found to exhibit nanotubes. Open red squares indicate populations with optically resolvable structures such as tubes and buds (with diameters above 0.5 µm) or GUVs without any defects. At least 15 GUVs were examined per population. The numbers indicate measured zeta potential values (std. dev. ± 10%). The experiments were limited by poor GUV yield at low voltages and temperature (rectangular area), and by water hydrolysis at high voltages and temperatures.

During hydration, the formed bilayers are not continuous over the whole surface but exhibit a high number of defects (such as membrane edges) which do not heal during the electroformation procedure^42,43^, see also SI Figure S9 and Movie S1. Defects drastically reduce the energetic barrier for lipid exchange between leaflets^44–46^, see Fig. 3a.

#### Electric field

An electric field will act on the charged lipids. Particularly, the electric field component in the direction normal to the bilayer will create a preference of charged lipids to one of the two leaflets depending on the electrode polarity^45^. The membrane is exposed to the externally applied potential and the resting potential that results from the surface charges on the ITO electrode. The static surface charges are essential because the time-averaged AC field vanishes on the timescale of GUV formation. At pH 7, ITO is *negatively* charged^47^, consistent with the location of the negatively charged lipids on the outer leaflet (facing away from the electrode), see Fig. 3a. To study the interaction between lipids and static surface charge, we varied the surface charges of the ITO plates by applying (static) electric DC-field, keeping all other conditions the same as in AC-electroformation, see Fig. 3b and Material and Methods. Note that GUV formation was found to be efficient only at *negatively* charged ITO electrodes. Presumably, adhesive interactions between the bilayers and a *positively* charged electrode^5,48^ prevent GUV swelling. We examined the prepared GUVs, assessed their tendency to form nanotubes after deflation and measured their zeta potential as a direct indicator of DOPG asymmetry. With increasing negative surface charge of the ITO electrode (higher DC field), the membrane asymmetry is increased (see zeta-potential values in Fig. 3b), which is fully consistent with the proposed mechanism. The results are also consistent with symmetric DOPG distributions in GUVs grown on PVA gels (see section above) in the absence of an external electrical field. Because of the polymer functional groups, the PVA-gel substrate exhibits a low surface charge.

#### Entropy counteracting lipid sorting

If the electric field created by the surface charges acts to relocate the charged lipids away from the ITO electrode, entropic forces should act against such a sorting. We thus systematically investigated the formation of asymmetric GUVs by varying both the surface charges of the ITO surface (as explained above) and the entropic forces by the electroformation temperature (Fig. 3b).

#### Different affinity of lipids to ITO substrate

In the stock solution and during the solvent evaporation after deposition on the ITO electrodes, lipids have a high degree of orientational freedom and assemble into bilayer according to their affinity to the ITO surface. In the absence of the applied external voltage, the surface potential of the electrode is negative. Presumably, the affinity (e.g. electrostatic attraction) of negatively charged lipids to the ITO surface is lower than that of neutral PC lipids leading to enrichment of charged lipids in the outer leaflet.

In this section we have performed electroformation with DC fields in order to isolate the effects on surface charge. AC-field electroformation is recommended for production of GUVs. As shown above at elevated temperature (60 °C, 2.2 V_rms_ AC-field) it results in almost symmetric DOPG distributions (Fig. 2). The increased temperature will act to reshuffle the lipids across the leaflets, while the increase of the AC-field amplitude should overcome the ITO offset voltage of about −0.8 V^47^, which will bring the time averaged surface charge closer to zero.

## Discussion and Outlook

The results presented here indicate that the conditions of electroformation of GUVs with charged lipids should be carefully scrutinized, especially when bilayer surface charge is important or the effects of membrane asymmetry are investigated. Membrane asymmetry was confirmed using quenching of a fluorescent DOPG analog, zeta-potential measurements, and annexin A5 binding to unmodified DOPS lipids. As we have shown, lipid distribution between bilayer leaflets depends on applied voltage, temperature, resting time after electroformation and potentially other factors such as electroformation time and maybe even thickness and topology of the initial lipid film. The electroformation protocol is not universal and all these parameters vary greatly between different groups and sometimes even within labs.

In view of the results reported here, electroformed (charged) vesicles should be left to rest for ~24 hours at room temperature if symmetric lipid distribution is required. To shorten this equilibration time, the flip-flop process can be accelerated by using higher temperatures and applied voltages during the electroformation. For electroformation at elevated temperatures and voltage, conditions should be carefully optimized and checked to avoid lipid oxidation. If the exact preparation conditions are not taken care of and not indicated in the reporting work in sufficient detail, it is hardly possible to understand any discrepancies between results reported from different labs. GUV-based assays for assessing curvature generation^16–18^ as discussed in the introduction should be rather sensitive to the vesicle preparation protocol, especially when the membrane is multicomponent and the different lipid species interact differently with the substrate used for growing the GUVs. As we demonstrated, charged lipids distribute asymmetrically and in this way, may influence the binding affinity of species in the solution. In a recent example, conflicting results on calcium-induced curvature generation in charged DOPC/DOPS GUVs have been reported^17,18,41^. As it appears from the published protocol, Simunovic *et al*.^17^ used vesicles right after preparation, while Graber *et al*.^18^ used GUVs over the time course of days. Our results for asymmetric distribution of the charged lipids corroborate the measured non-zero (negative) spontaneous curvature for freshly prepared DOPC/DOPS vesicles in the absence of calcium ions^17^. We speculate that the different vesicle surface charges and non-zero spontaneous curvature in the absence of calcium ions in^17,18^ could be one of the main reasons for the opposing findings for calcium-generated curvature. We thus emphasize the importance of considering lipid asymmetry resulting from the different electroformation protocols for charged GUVs. Additionally, the correct interpretation of measured isotherms for protein binding to charged GUVs might be hindered if the enrichment of charged lipids on the outer membrane is not taken into account. An example for a change in the binding affinity of the protein annexin A5 to DOPS-doped membranes was demonstrated here as vesicles were left to equilibrate and leaflet asymmetry diminished. Protein binding might be dependent on membrane curvature^49^ and, thus, further experimental studies are needed to distinguish the different contributions.

Membrane asymmetry and appearance of nanotubes does not have to be considered as an undesirable consequence. In fact Barooji *et al*.^19^ reported binding of BAR proteins to spontaneously tubulated DOPC:DOPS GUVs, which were prepared via electroformation. We believe that spontaneous tubulation proceeded via the mechanisms discussed here. In general, the ability to precisely tune the composition of lipid bilayer leaflets by an external electric field can be exploited in the assembly of cell-like compartments. Cellular lipid membranes are mostly asymmetric in their lipid distribution and PS asymmetry is important for cellular functions^50^. Preparation of asymmetric GUVs by electroformation offers an alternative to the protocol of lipid exchange assisted by cyclodextrin molecules or microfluidic assembly of GUVs^51,52^. Potentially, not only lipids but also charged macromolecules embedded in bilayers could be orientated by finely tuning the electroformation conditions. When the generation of membrane asymmetry is desired, one should be aware that lipid-flop flop is a continuous process and as such will gradually diminish asymmetry with time. The rather slow flip-flop rates of typical phospholipids should allow for stable generation of membrane asymmetry on the timescale of a few hours.

Finally, we demonstrated that lipid-only membranes can undergo morphological changes (budding and tubulation) as a result of asymmetric lipid distribution between their leaflets. All biomembranes exhibit such a compositional asymmetry. Curvature generation relying on the present lipid asymmetry offers cells an energetically cheap and protein-free pathway for modulating organelle shapes.

## Materials and Methods

### Electroformation of GUVs

Chloroform stock solutions of 1,2-dioleoyl-sn-glycero-3-phosphocholine (DOPC) and 1,2-dioleoyl-sn-glycero-3-phospho-(1′-rac-glycerol) (sodium salt) (DOPG) were mixed at a molar ratio of 8/2 with a final lipid concentration of 4 mM. The lipids were obtained from Avanti Polar Lipids (Alabaster, AL). At neutral pH, DOPG contributes a negative surface charge to the vesicles. Unless stated otherwise, 1 mol% of the fluorescent tail-labeled dye NBD-PG, i.e., 1-oleoyl-2-[12-[(7-nitro-2-1,3-benzoxadiazol-4-yl)amino]dodecanoyl]-sn-glycero-3-[phospho-rac-(1-glycerol)] (ammonium salt) (Avanti Polar Lipids) was added. In total, 10 µL of the stock solution were spread on two conductive ITO-coated glasses (ITO film thickness <100 nm, resistance 50 Ω; Präzisions Glas & Optik, Iserlohn, Germany). To eliminate trace amounts of chloroform, the glasses were kept for 2 to 2.5 hours under vacuum at room temperature (RT). They were then assembled together with a Teflon spacer to form a chamber of 2 mL volume that was filled either with a buffered sucrose solution (17 mg/ml sucrose, 2 mM HEPES pH 7.4, containing 1 mM EDTA, at total osmolarity of 63 ± 2 mOsmol/L) or with a buffer only (2 mM HEPES pH 7.4, containing 1 mM EDTA, osmolarity 8 ± 2 mOsmol/L). All chemicals were obtained from Sigma. Unless stated otherwise, electroformation was carried out with a sinusoidal voltage of 10 Hz with an amplitude of 630 mV root mean square (RMS; all values in the text are given in RMS) for 2.5 hours. Note that we measure and report the voltage effective at the ITO glasses and not the voltage set by the function generator, which was 1 V peak-to-peak at 50 Ω load. In some cases, static voltage (direct current, DC) was applied as described in the main text, while keeping the rest of the conditions identical to those for AC-field electroformation. For growing vesicles at temperatures higher than RT, the whole chamber was placed inside an oven. The vesicles were harvested from the chamber and either used immediately or left to rest for 26 hours at RT in the dark. For GUV growth on platinum wires, 5 µL of 0.4 mM DOPC/DOPG lipid solution in chloroform were spread on two 2 cm long platinum wires (wire to wire distance was 5 mm which is the same as the inter-electrode distance when using ITO plates). The electroformation voltages were adjusted to yield the same field strength effective at the platinum wire clamps as for experiments on ITO electrodes.

Experiments with vesicles containing 1,2-dioleoyl-sn-glycero-3-phospho-L-serine (sodium salt) (DOPS) and DOPC were performed similarly to those with DOPC/DOPG membranes. GUVs from lipid mixtures of varying DOPS content as indicated in the main text were grown in 300 mM sucrose solution buffered with 2 mM Hepes pH 7.4.

### Gel-assisted growth of GUVs

GUVs were grown on a polyvinyl alcohol (PVA) film as first described in ref.^22^. Briefly, 50 µL of 5% w/v PVA (Merck) solution in water was spread on a cleaned (rinsed in ethanol and double distilled water) glass slide to form a thin film. The PVA film was dried at 50 °C for 30 minutes. 5 µL of 2 mM lipid solution in chloroform was spread on the PVA film and the solvent was evaporated in vacuum for 2.5 hours. Using a Teflon spacer, a chamber of about 700 µL volume was assembled and lipid films were hydrated in the same buffered sugar solution as used in the electroformation method. After swelling for 30 minutes, GUVs were harvested and used right away.

### GUV deflation and imaging

One part of the vesicle suspension was mixed with a slightly hypertonic (1% higher) buffered glucose solution with a volume ratio of 1/2 vesicle suspension to diluting solution (to a final volume of 90 µL). Before imaging, the vesicles were deflated by water evaporation at RT for 40 minutes. The gradual and steady increase in osmolarity (to a value of approximately 88 mOsmol/L, as measured by the decrease in mass during evaporation) allowed the vesicles to deflate in a smooth manner. In some experiments, the vesicle membrane was left unlabeled and a water soluble dye (sulforhodamine B, Sigma) of 2.5 µM concentration was added to the outside solution.

Imaging was performed on a Leica confocal SP5 setup (Mannheim, Germany). Sulphorhodamine was excited with a 561 nm laser (diode-pumped solid-state laser) and NBD-PG was excited using the 488 nm line of an Argon laser. The fluorescence signals were collected in the ranges 570–650 nm and 494–642 nm, respectively. For out-of-focus imaging of the nanotubes, the confocal pinhole was sometimes opened from 1 to 2 Airy units. To prevent vesicle adhesion, cover glasses were coated with 10 mg/ml bovine serum albumin (BSA, Sigma) in pure water solution for 15 minutes prior to usage. Prior to depositing the vesicle suspension on the cover glasses, the latter were rinsed in pure water to remove any unbound BSA.

### Quenching assay and fluorescence intensity measurements

NBD-PG located in the outer leaflet of the vesicle membrane was quenched by reduction with dithionite^53^. For each experiment, we first prepared a fresh 1 M stock solution of sodium dithionite (Sigma) in the sucrose buffer used for vesicle preparation. Then, 1 µL of this quenching buffer was pipetted into 100 µL of vesicle suspension in sucrose buffer. The solution was gently stirred to ensure homogenous distribution of the quenching agent. The vesicles were then incubated for 15 minutes and consecutively diluted in a slightly hypertonic buffer, in the same manner as with vesicles that were not treated with the quenching agent. For quantification of quenching experiments, the vesicles were not left to deflate via evaporation of the outside solution. GUVs prepared in this way appeared spherical and had no internal tubes. On the timescales of the observations, we did not observe any drift in GUVs fluorescence values due to leakage of quenching agent into the interior of the vesicle. For each population, at least 20 GUVs were imaged at the equatorial plane. Imaging settings were maintained between experiments, e.g. detector gain, laser power, pixel dwell time and resolution. Image analysis was used to estimate the fluorophore concentration of the acquired images. Using ImageJ^54^ the image was first convoluted with a Gaussian filter of kernel size equal to 2 pixels (corresponding to 240 nm). A typical image is shown in Figure S5. Then, to evaluate the fluorophore concentration, the maximum pixel intensity value of each vesicle was measured. We have also checked a limited set of GUVs with a more established method of fluorescent quantification, namely, line profiles across the equator of a confocal section, and found similar results. We checked for possible artefacts related to dye degradation during overnight storage of the GUVs (kept in the dark). We found no significant change in overall brightness and in dye distribution between GUVs, see Figure S6. We have already shown that the bending rigidity of DOPC/DOPG vesicles does not change significantly after 26 hours of storage, which indicates lack of lipid degradation^48^. Bending rigidity of 20 k_B_T ± 1 k_B_T was measured via fluctuation spectroscopy following the approach reported in ref.^55^.

### Zeta potential measurements

We have conducted zeta potential measurements on GUVs following the approach of Carvalho *et al*.^29^ using a ZetaSizer Nano ZS (Malvern, UK) instrument. For zeta potential measurements we used 600 µL of a GUV suspension directly pipetted from the electroformation chamber (symmetric sucrose conditions). We conducted no more than 4 measurements on each sample. The voltage for electrophoretic movement was set to 150 V in the instrument settings.

Because electrophoretic vesicle movement is measured by light scattering, we have some reservation of the validity of the data for GUV suspensions. Vesicle sizes within the sample are polydisperse and it is known that in heterogeneous samples light scattering only gives a misleading quasi-average value across the population. Additionally, (charged) GUVs are unstable in electric field pulses, as applied by the instrument. This was evidenced by decrease of the signal intensity with repeated measurements on the sample. To find a benchmark for a valid measurement, we considered the output of the zeta potential measurements instrument (Malvern ZetaSizer) referred to as “quality report”. Only measurements on populations fitting to the instruments quality criteria were considered. This lead to discarding some of the data for conditions which suffered from low light count due to the highly dilute samples (low voltages) and very polydisperse populations (high temperature) and GUV grown on PVA (lipid or PVA aggregates sedimenting in solution). Despite these limitations, Carvalho *et al*.^29^ has shown a clear correlation between measured zeta potential of GUV samples and liposomes preparations of uniform size (200 nm diameter). We therefore assume that the relative trends between samples are a valid measure.

### Annexin A5 binding assay

Annexin V CF594 fluorescent conjugate (Annexin A5) was obtained from Biotium (Cat. No. 29011). GUV were grown in 300 mM sucrose solution as described above and subsequently diluted 1:10 in a buffer containing 150 mM NaCl, 10 mM HEPES, and 2 mM CaCl_2_. Annexin A5 was incubated with the diluted GUV solution at a final concertation of 5 μg/ml for 20 minutes at room temperature. GUVs were imaged on a Leica SP8 confocal microscope using the 552 nm laser line (solid state laser) and fluorescence was collected between 575 and 675 nm. Image quantification was performed as described above for the NDB quenching experiments.

## Electronic supplementary material

Supporting data

supporting movie
