# Supplementary material for: Charged giant unilamellar vesicles prepared by electroformation exhibit nanotubes and transbilayer lipid asymmetry
Source: Sci Rep. 2018 Aug 7;8:11838. doi: 10.1038/s41598-018-30286-z (PMC6081385; doi:10.1038/s41598-018-30286-z)
Supplement: Supplementary file 1 — Supporting data [file 41598_2018_30286_MOESM1_ESM.pdf]

Supporting Information

Charged giant unilamellar vesicles prepared by electroformation exhibit nanotubes and transbilayer lipid asymmetry

*Jan Steinkühler[1], Philippe De Tillieux[1,2], Roland L. Knorr[1], Reinhard Lipowsky[1], Rumiana Dimova[1]**

[1] Department of Theory and Bio-Systems, Max Planck Institute of Colloids and Interfaces, Science Park Golm, 14424 Potsdam, Germany
[2] Current address: Department of Electrical Engineering, Polytechnique Montreal, Montreal, Quebec, Canada, H3T 1J4

* Corresponding author: dimova@mpikg.mpg.de

	Total number of vesicles	Vesicles with inward structures, %	Vesicles without defects, %	Vesicles with outward structures, %
DOPC/DOPG, 1h	27	**70.4**	14.8	14.8
DOPC, 1h	32	15.6	56.3	28.1
DOPC/DOPG, 26h	29	20.7	**69.0**	10.3
DOPC, 26h	27	18.5	77.8	3.7

Table S1. Electroformed vesicles in buffer (no sugars). Number of observed GUVs with inward structures (such as buds, tubes/pearl chains), defect free and with outward structures. Outward structures are often single long nanotubes and presumably result from the detachment of vesicles from the electrode surface in the electroformation chamber. Strong shifts in DOPC/DOPG vesicles morphologies after storage are indicated in bold. The observations were performed using a 40x objective with 0.6 NA under epifluorescence microscopy. Only vesicles with diameter larger than 10 µm were considered.

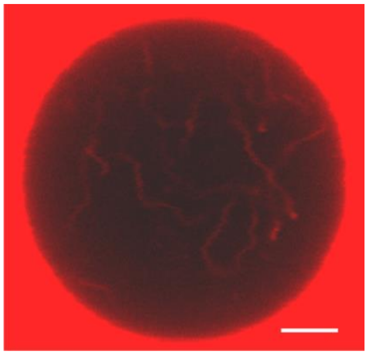

Figure S1. DOPC/DOPG (8/2) GUV deflated in sucrose / glucose solution**.** The nanotube interior is accessible to sulphorhodamine (red) dissolved in the solution outside the GUVs. Here the GUV membrane was not stained. Scale bar indicates 5μm.

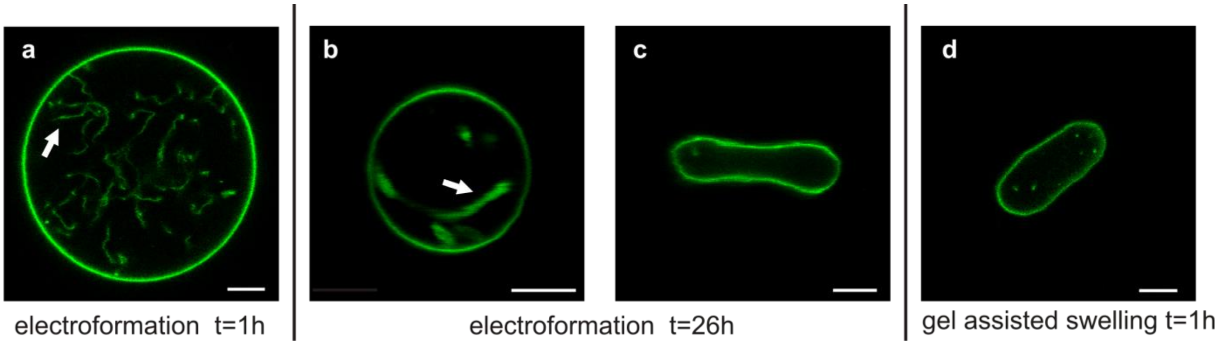

Figure S2. Typical morphologies of different DOPC/DOPG vesicle populations obtained by confocal microscopy (cross sections). All vesicles were left to deflate in sucrose/glucose solutions as indicated in the SI Material and Methods (a) Electroformed vesicle 1 hour after preparation. (b, c) Vesicles after 26 h storage at RT. (d) A GUV formed by the gel-assisted method right after preparation. Arrows point to lipid tubes. All scale bars 5 µm.

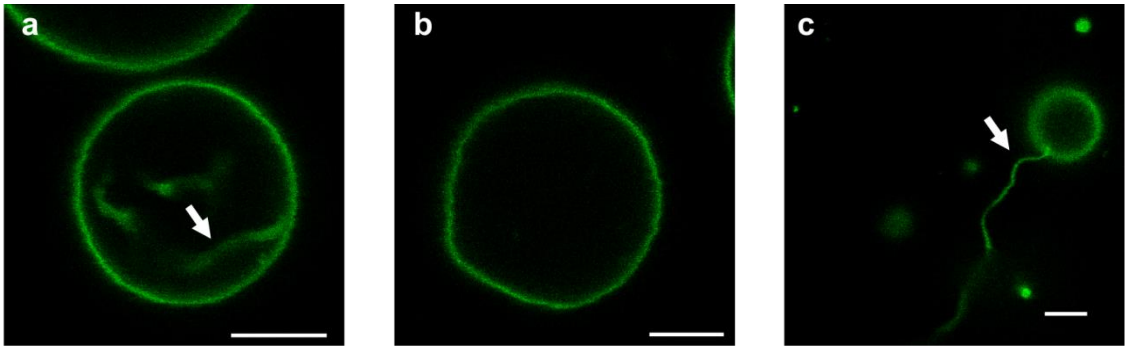

Figure S3. GUVs grown on PVA exhibit a heterogeneous response upon deflation in sucrose/glucose solutions: Whereas some vesicles undergo tubulation and/or budding either inward (a) or outward (c) and the tubes/buds are of varying diameter, other GUVs show no such defects (b). Scale bars indicate 5μm.

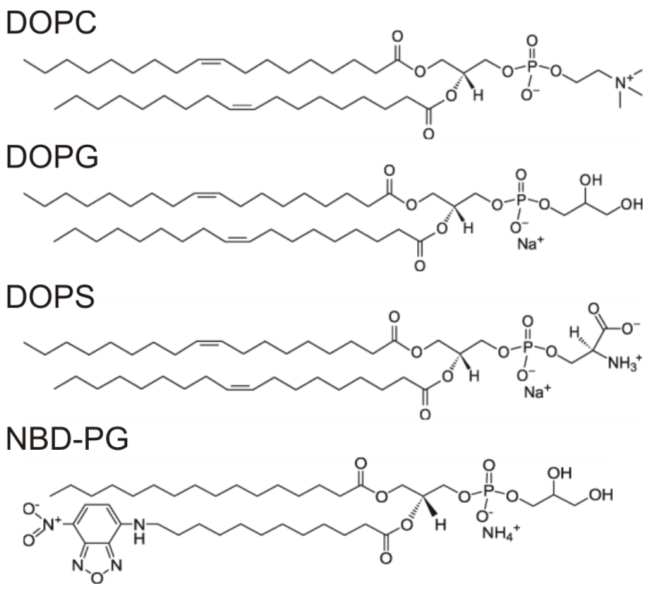

Figure S4. Lipids and lipid analogs used in this work.

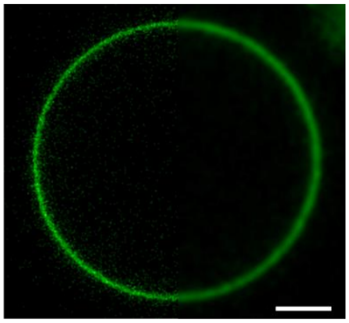

Figure S5. Typical image of a vesicle used for quantification of the fluorescence quenching assay as obtained by confocal microscopy. The right half of the vesicle is shown after Gaussian filtering. Scale bar indicates 5 μm.

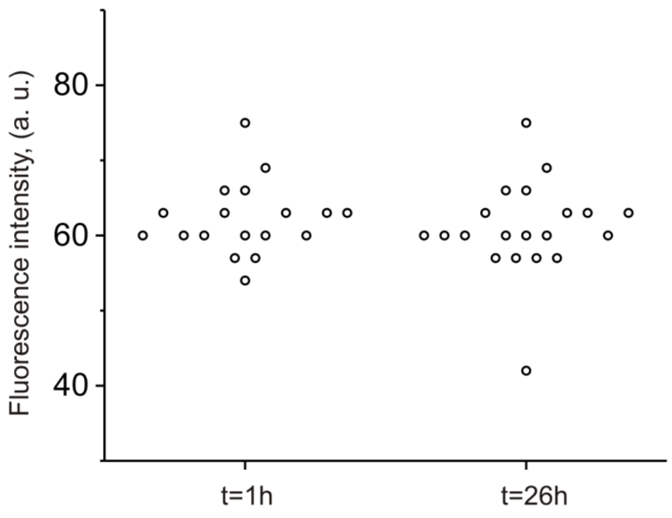

Figure S6. Fluorescence intensities of electroformed DOPC/DOPG GUVs one hour after preparation and after 26-hour storage. Every point represents a measurement on an individual vesicle. No significant difference in the two populations is observed, indicating no degradation of the used dye (NBD-PG) during storage.

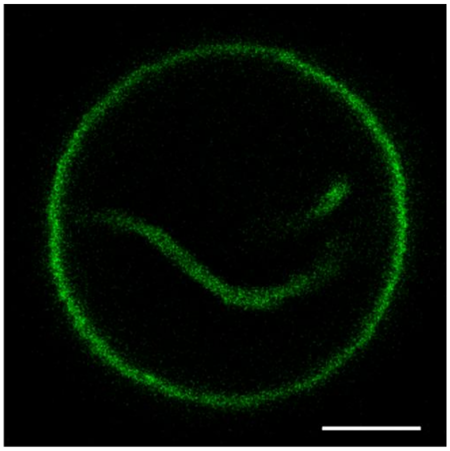

Figure S7. Confocal image of a DOPC/DOPG GUV grown on platinum wires (as described in the Material and Methods) and osmotically deflated right after preparation. The majority (>90%) of the GUVs exhibit inward pointing tubes. In some cases, GUVs appear to exhibit thicker tubes, with diameter closer to the optical resolution when compared to the morphologies of GUVs grown on ITO, see Figure S2a. Presumably differences in direct platinum-lipid and lipid film morphology (as films are deposited on cylindrical wires or a flat surface), could play a role here. These effects were not investigated further. Scale bar indicates 5μm.

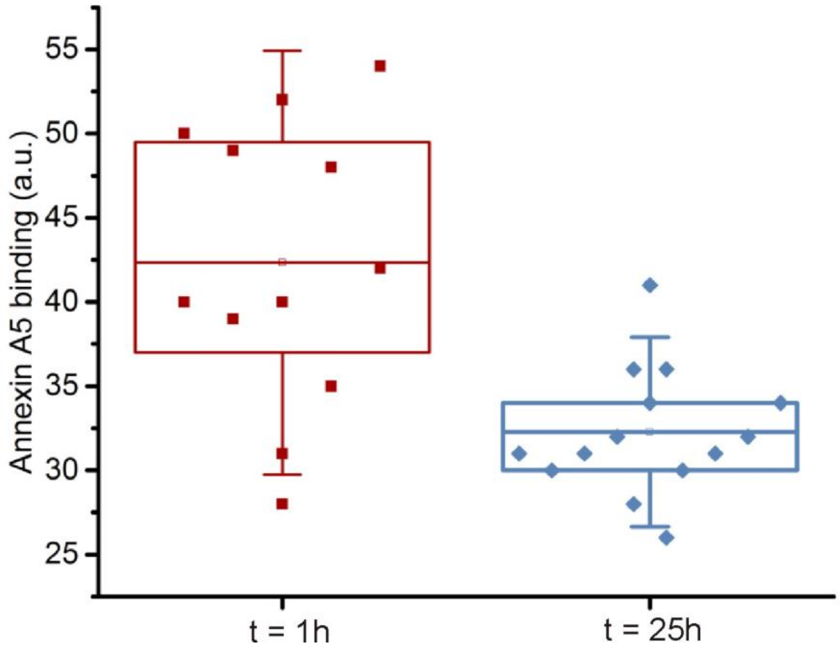

Figure S8. Binding of fluorescently labeled annexin A5 to DOPC GUV doped with 5 mol% DOPS as quantified from confocal microscopy of the vesicles 1 h and 25 h after preparation. Annexin A5 binding is significantly reduced from 42 ± 8 std. dev. intensity units (n=12) to 32 ± 4 std. dev. (n=14) ($p<0.005$, Student t-test). Each data point represents one vesicle.

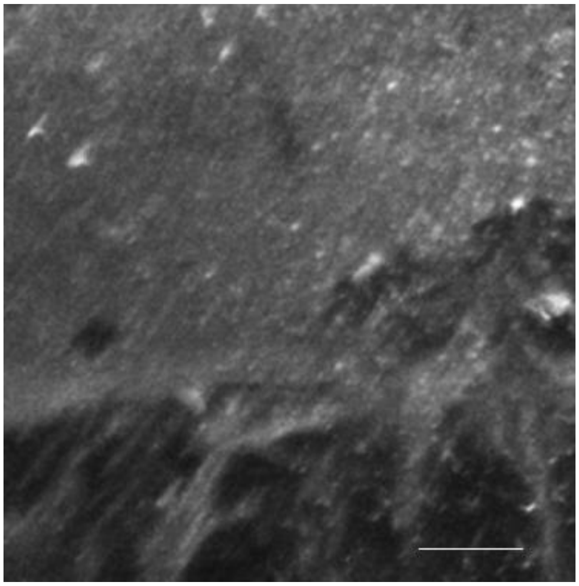

Figure S9. Maximum projection image of the lipid film on ITO after hydration in sucrose buffer (obtained by confocal microscopy). Scale bar 50 µm. The fluorescent signal is proportional to the number of bilayers. Thus, variations in the gray values indicate edges of individual bilayers. See also Movie S1.

Movie S1. Large scale bilayer defects do not heal on the timescale of GUV growth. The movie shows time series of DOPC/DOPG GUV AC-field electroformation at 10Hz and 630 mV RMS applied voltage, room temperature, maximum projection obtained from confocal images at the electrode. Scale bar indicates 100 µm. Counter indicates hours:minutes since start of voltage application.
